# Transmembrane TNF-α as a Novel Biomarker for the Diagnosis of Cytokine Storms in a Mouse Model of Multiple Organ Failure

**DOI:** 10.1007/s10753-022-01738-6

**Published:** 2022-09-14

**Authors:** Peng Yang, Yimin Zeng, Fang Yang, Xin Peng, Yongsheng Hu, Xuhong Tan, Ruping Zhang

**Affiliations:** Department of Clinical Laboratory, The Second Affiliated Hospital, Guizhou University of Traditional Chinese Medicine, Guiyang, 550001 China

**Keywords:** Multiple organ failure, Transmembrane TNF-α, Inflammatory responses, Cytokine storm, Diagnostic marker

## Abstract

A cytokine storm (CS) is an out-of-control inflammatory response closely associated with the progression of diseases, such as multiple organ failure (MOF), severe sepsis, and severe or critical COVID-19. However, there is currently a lack of reliable diagnostic markers to distinguish CS from normal inflammatory responses. Tumor necrosis factor-α (TNF-α) includes transmembrane TNF-α (tmTNF-α) and secreted TNF-α (sTNF-α). The MOF mouse model in this study showed that the tmTNF-α expression changes in the neutrophils differed from the serum TNF-α and serum IL-18, INF-γ, IL-4, and IL-6. Furthermore, tmTNF-α, instead of serum TNF-α, IL-18, INF-γ, IL-4, and IL-6, reflected liver and kidney tissue damage and increased with the aggravation of these injuries. Analysis of the ROC results showed that tmTNF-α effectively distinguished between inflammatory responses and CS and efficiently differentiated between surviving and dead mice. It also significantly improved the diagnostic value of the traditional CRP marker for CS. These results indicated that the tmTNF-α expressed in the neutrophils could be used to diagnose CS in MOF mice, providing an experimental basis to further develop tmTNF-α for diagnosing CS patients.

**Supplementary Information:**

The online version contains supplementary material available at 10.1007/s10753-022-01738-6.

## INTRODUCTION

A cytokine storm (CS) is an uncontrolled systemic inflammatory response involving elevated circulating cytokine levels and immune cell over-activation, which can be triggered by treatment, pathogenic infections, cancer, and autoimmune diseases [1, 2]. Although the factors driving CS may differ, late-stage clinical manifestations converge and often overlap [3].Under normal stress, the inflammatory response mediated by increased cytokines helps control infection, which is beneficial for the body to maintain the stability of the internal environment [4]. However, uncontrolled inflammatory responses can mediate CS that can cause severe damage to the host, including acute respiratory distress syndrome, kidney failure, acute liver injury, multiple organ failure (MOF), and even death [2]. Therefore, accurately identifying inflammatory responses and CS is vital for treating patients with associated diseases using selective anti-inflammatory therapies.

With the continuous outbreak of the Ebola virus, avian influenza, SARS coronavirus, and novel Coronavirus in recent years, the role of CS in patients with sepsis, MOF, and severe pneumonia has become a significant concern [5, 6]. Defining the normal inflammatory response and CS have attracted considerable attention for preventing and treating severe patients. The tumor necrosis factor-α (TNF-α), interleukin-18 (IL-18), interleukin-6 (IL-6), interferon-γ (IFN-γ), interleukin-4 (IL-4), and interleukin-10 (IL-10) cytokines represent the key mediators driving CS. Therefore, clinical attempts have been made to distinguish CS by detecting cytokine levels [7]. Due to the short half-life of cytokines, the changes in the cytokine content are not completely consistent with the severity of the disease. It is difficult to distinguish a CS from an inflammatory response by detecting cytokine levels in the systemic circulation. Furthermore, it cannot accurately reflect the severity of the disease or confirm the diagnostic threshold of a CS [5]. In recent years, some studies have shown that the content changes in non-specific inflammatory markers, such as C-reactive protein (CRP), are related to the severity of the disease and can be used for the auxiliary detection of CS [8, 9]. However, the sensitivity and specificity of these markers still do not meet the needs for the clinical diagnosis and treatment of CS.

TNF-α is divided into transmembrane TNF-α (tmTNF-α) and soluble TNF-α (sTNF-α). tmTNF-α is a precursor of sTNF-α, expressed as 26kd on the cell membrane and cleaved to sTNF-α (blood TNF-α) via the TACE enzyme [10]. However, the role of tmTNF-α in disease development differs from sTNF-α. Moreover, tmTNF-α is often difficult to detect or can only be determined at low levels, even upon stimulation, due to being cleaved chiefly by the TNF-α converting enzyme [11]. Several studies have shown abnormally high tmTNF-α expression in the neutrophils during acute inflammation [12], non-small cell lung cancer [13], and primary breast cancer [14]. Therefore, the role of tmTNF-α in diseases has attracted increasing attention. This study aims to elucidate the diagnostic value of tmTNF-α in CS involving MOF.

## MATERIALS AND METHODS

### Experimental Animals

The animal study was approved by the Animal Care and Use Committee of Guizhou University of Traditional Chinese Medicine. Six- to 7-week-old male C57BL/6 mice were purchased from Sibefu Biotechnology (BeiJin, China). The animals were housed in pathogen-free conditions and allowed free access to water and food.

### Animal Model Preparation

Liver failure was induced via intraperitoneal injection with 10 μg/kg LPS (Sigma-Aldrich; *Escherichia coli* 0111: B) and 500 mg/kg D-gal (Sigma-Aldrich). Liver tissue and blood samples were then collected before (0 h) and after (2 h, 4 h, and 6 h) injection.

#### Flow Cytometry (FCM) Analysis

Here, 50 ML of blood was incubated at room temperature for 40 min with 5μL of a PE-conjugated anti-mouse TNF-α antibody, after which 300μL stromatolyser-4DL FFD-201A was added for 5 min, followed by 500μL PBS. The mixture was centrifuged at 2500 g for 5 min, after which the supernatant was discarded, and 300μL PBS was added for resuspension. The stained cells were analyzed using an LSR II flow cytometer (Becton Dickinson, San Jose, CA, USA). The cytometric data were analyzed using FlowJo software. The expression levels of tmTNF-α in the neutrophils and peripheral blood mononuclear cells (PBMCs) were analyzed after removing the adherent cells and a small number of dead cells. All the reagents involved in FCM were purchased from BD Biosciences.

### Enzyme-linked Immunosorbent Assay (ELISA)

The TNF-α, IL-18, INF-γ, IL-6, IL-4, and CRP levels were measured using commercial ELISA kits (HePeng Biological) according to the protocols of the manufacturer. Briefly, the plasma and standard were transferred to an ELISA plate and mixed for 1 h at 37 °C. After washing, enzyme-labeled antibody working liquid was added to each well for 30 min at 37 °C. Subsequently, the reaction was terminated using a termination solution, and the absorbance of each well was measured at a wave length of 450 nm. The sample content was determined via a standard curve, which consisted of the concentration gradient of the standard substance on the *X*-axis plotted against the optical density value on the Y-axis.

### Histology

The liver and kidney tissues were fixed with 10% formalin for 24 h and then embedded in paraffin wax. The tissue Sects. (4 μm) were routinely stained with hematoxylin–eosin (H&E) for morphological evaluation.

### The Detection of Liver and Renal Function

The ALT and AST levels of the creatinine (Cr) and cystatin C (CysC) were measured using a Mindray ExC8100 system in the clinical laboratory of the Second Affiliated Hospital, Guizhou University of Traditional Chinese Medicine.

### Statistical Analysis

The data were shown as the median with standard deviation. The differences between the two groups were assessed via Mann–Whitney tests(unpaired tests). A value of *P* < 0.05 was considered statistically significant. An AUC was used to sensitively and specifically determine the predictive values of the tested biomarkers to discriminate between the mice with liver failure (6 h after injection) and the HC group and between the mice displaying an inflammatory reaction and CS. All statistical analyses were performed using GraphPad Prism V9 software.

## RESULTS

### The tmTNF-α Expression Level in the Neutrophils was Significantly Higher in Mice with MOF

The expression level of tmTNF-α in the peripheral blood leukocytes of a mouse model involving acute liver failure with multiple organ damage was evaluated to clarify the role of tmTNF-α in CS involving MOF. As shown after H&E staining of the liver sections, obvious sinus congestion, liver cord rupture, necrosis, and inflammatory cell infiltration were observed 6 h after LPS/D-GAL injection (Supplementary Fig. 1a), and the serum ALT and AST levels were significantly elevated (Supplementary Fig. 1b). Similarly, the renal tissue and renal function showed similar changes, with tubular epithelial cell necrosis and inflammatory cell infiltration (Supplementary Fig. 2a), while the serum Cr and CysC content was also significantly increased (Supplementary Fig. 2b).

**Fig. 1:**
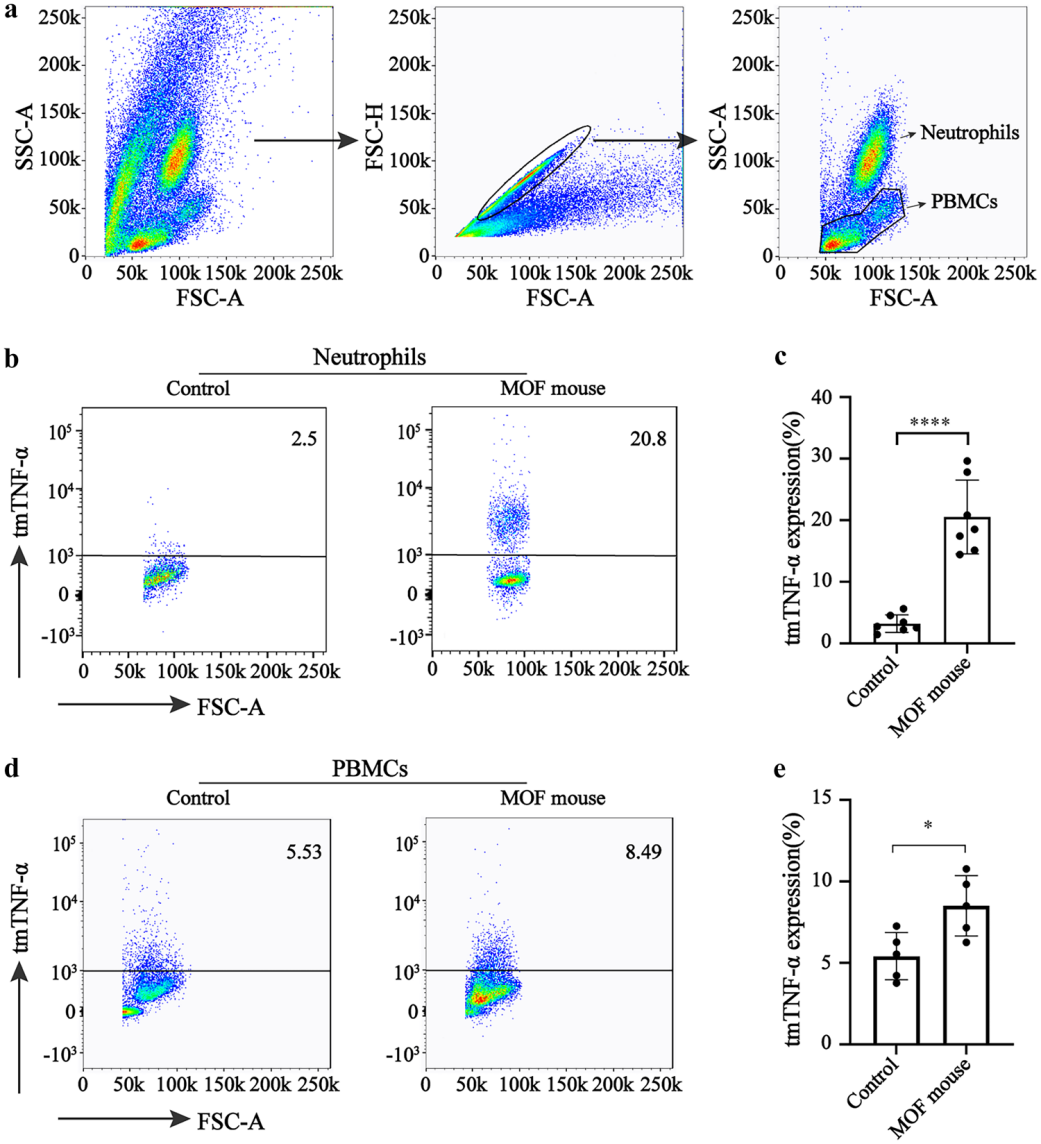
The expression level of tmTNF-α in the neutrophils and PBMCs in the blood of the MOF control mice. **a** A representative FCM of the leukocyte grouping in the peripheral blood of the mice. **b** A representative FCM image of the tmTNF-α in the neutrophils in the mouse blood. **c** Quantitative analysis of the tmTNF-α in the neutrophils (*n* = 7). **d** A representative FCM image of the tmTNF-α in the PBMCs of the mouse blood. **e** Quantitative analysis of the tmTNF-α in the PBMCs (*n* = 5). The MOF mice were compared with the control mice ^*^Indicates *P* < 0.05 and ^****^indicates *P* < 0.0001.

According to the FCM analysis results, the leukocytes in the peripheral blood of the mice displayed three typical cell populations (Fig. 1a). The expression levels of the tmTNF-α in the neutrophils and peripheral blood mononuclear cells (PBMCs) of the MOF mice blood were examined to further demonstrate the role of tmTNF-α in the pathogenesis of the CS involving MOF. The results showed that the expression of tmTNF-α in the peripheral blood neutrophils of the MOF mice was 21.6%, which was significantly higher than that of the control mice, *P* < 0.00001 (Fig. 1b, c). However, the expression of tmTNF-α in the PBMCs, including lymphocytes and monocytes, was only 7.7%, which was slightly higher than that of the control mouse, *P* < 0.05 (Fig. 1d, e). These results suggest that higher tmTNF-α inneutrophils may play an important role in the development of CS involving MOF.

### The tmTNF-α Expression Changes in the Neutrophils Differed from the Serum Cytokines in the MOF Mouse Model

**Fig. 2:**
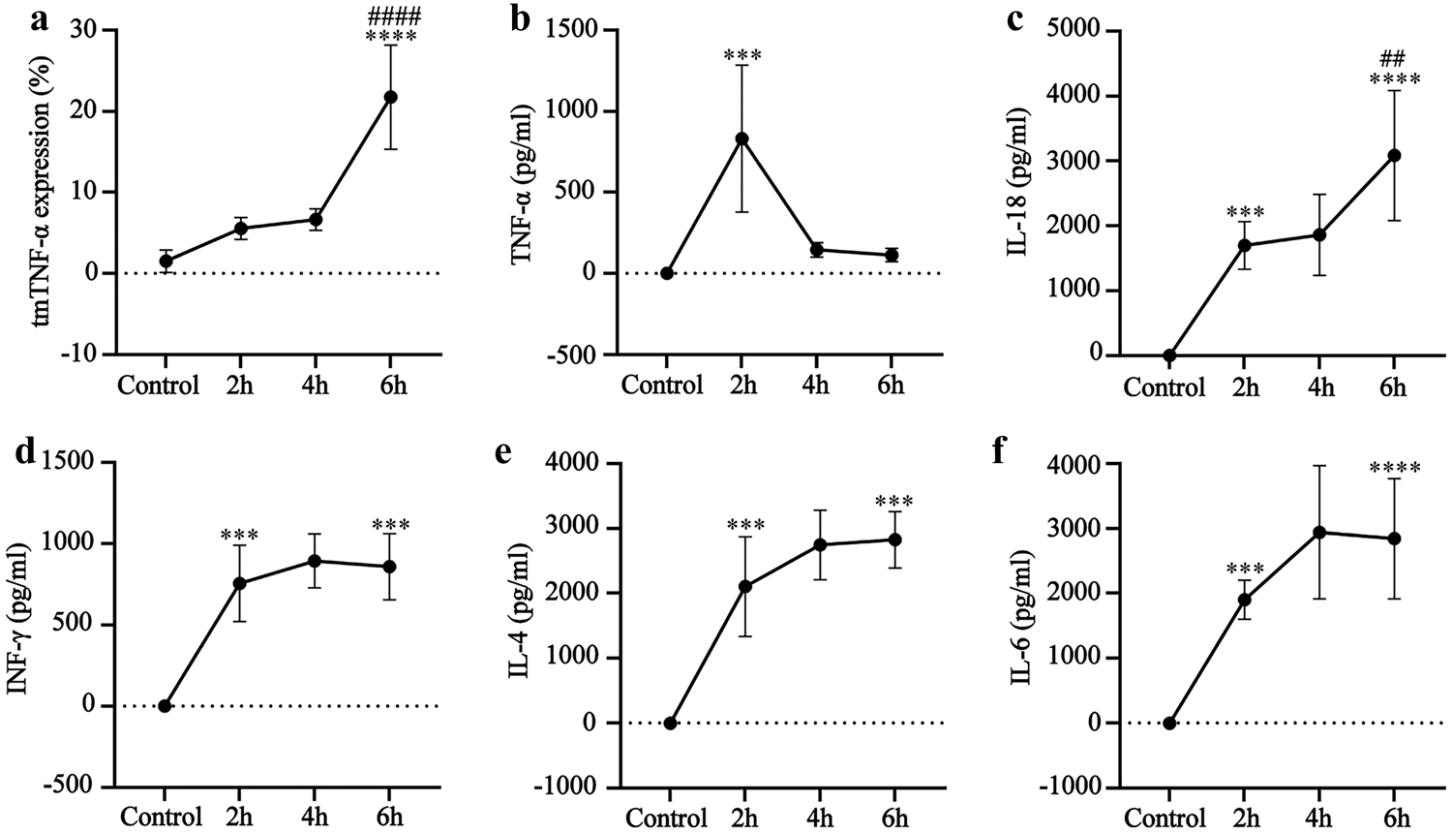
The dynamic tmTNF-α expression changes in the neutrophils and serum cytokines in the mice with MOF. **a** The tmTNF-α expression changes in the peripheral blood neutrophils. The changes in **b** TNF- α, **c** IL-18, **d** INF- γ, **e** IL-4, and **f** IL-6 content at different time points after LPS/D-gal injection (*n* = 7). The control group was compared with the treatment group at different times after LPS/D-gal injection. ^*^Indicates *P* < 0.05, ^***^indicates *P* < 0.001, and ^****^indicates *P* < 0.0001. In a comparison between treatment groups 4 h and 6 h after LPS/D-gal injection, ^##^indicates *P* < 0.01, and ^####^indicates *P* < 0.0001.

This study showed that the dynamic tmTNF-α expression changes in the MOF mouse model differed from the serum cytokines. At 2 h and 4 h after LPS/D-gal injection, the tmTNF-α expression in the neutrophils did not display a substantial increase but was significantly higher after 6 h, with a value of *P* < 0.0001 compared with the control group and *P* < 0.001 compared with the 4 h sample group (Fig. 2a). Furthermore, the tmTNF-α expression changes were consistent with those denoting liver tissue damage and liver function and increased with aggravated liver tissue damage (Supplementary Figs. 1a, b). They also corresponded with kidney tissue damage and renal function changes (Supplementary Figs. 2a, b). The changes in the serum TNF-α levels were not associated with liver and kidney tissue injury and peaked 2 h after injection while decreasing to normal levels 6 h after treatment (Fig. 2b). The other serum cytokines, IL-18, INF-γ, IL-4, and IL-6, were significantly higher 2 h after injection, reaching the highest levels at 4 h or 6 h after injection. However, no significant changes were evident in the other cytokines, except for IL-18 6 h after injection compared with 4 h (Fig. 2c, d, e and f). These content changes were in consistent with those in liver and kidney tissue injury. These results suggest that tmTNF-α displays a higher value than serum cytokines for diagnosing CS.

### The tmTNF-α Expression Level in The Dead Mice Differed from the Surviving Mice

**Fig. 3:**
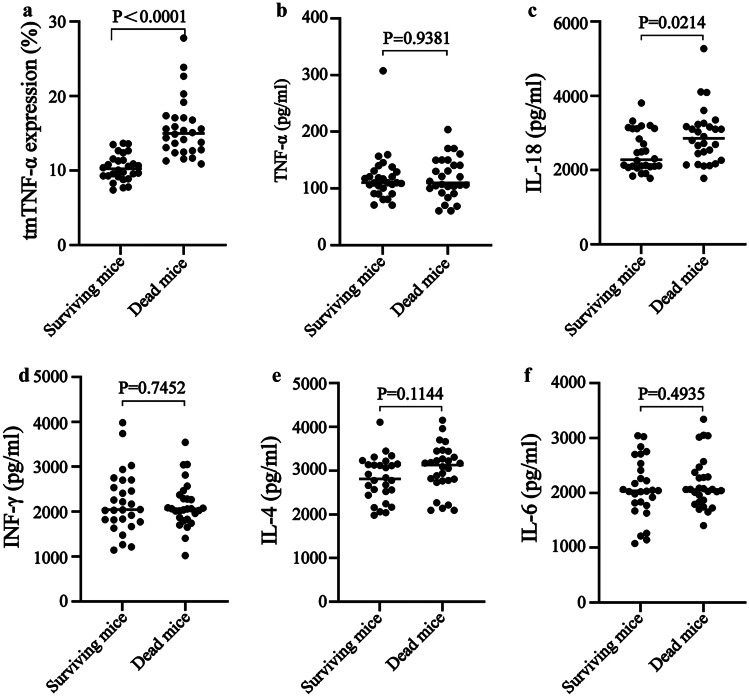
The tmTNF-α expression levels in the neutrophils and serum cytokines in the dead and surviving mice. **a** The tmTNF-α expression level in the peripheral blood neutrophils. The **b** TNF-α, **c** IL-18, **d** INF-γ, **e** IL-4, and **f** IL-6contentinthe dead (*n* = 28) and surviving mice (*n* = 28).

At 6 h after LPS/D-gal injection, 32 of the 60 mice died, while the survival rate was 46.67%. To further examine the relationship between tmTNF-α and the disease severity caused by CS, the tmTNF-α expression differences between the surviving and dead mice were examined, while the TNF-α, IL-18, INF-γ, IL-4, and IL-6 differences between the two groups were also compared. The results showed that the tmTNF-α expression level in the dead mice was significantly higher than in the surviving mice (*P* < 0.0001; Fig. 3a). Except for IL-18, TNF-α, INF-γ, IL-4, and IL-6, no significant differences were evident between the serum cytokine levels of the two groups (Fig. 3b, c, d, e, and f), suggesting that the tmTNF-α expression level could distinguish the disease severity caused by CS.

### The Evaluation of the Diagnostic Value of tmTNF-α and IL-18 for CS in MOF Mice

**Fig. 4:**
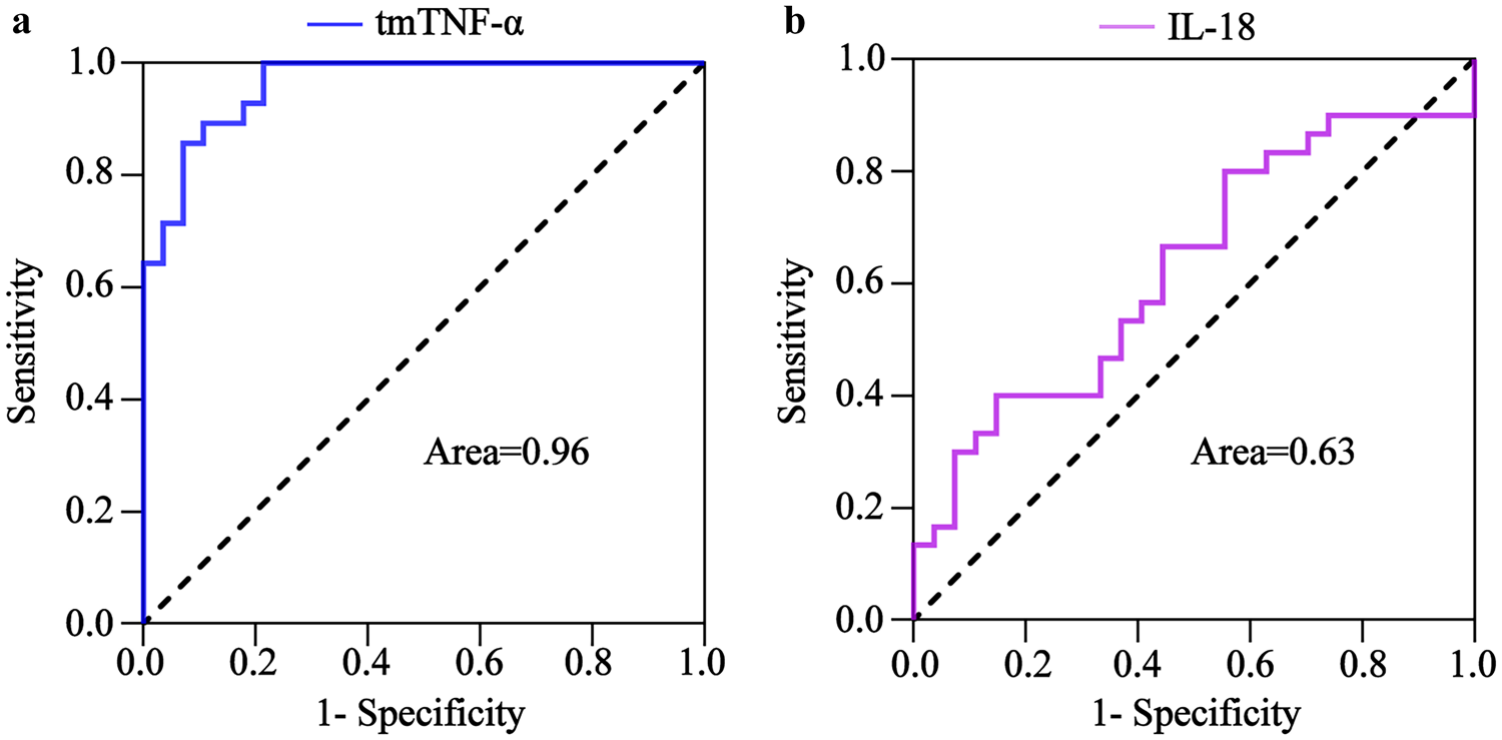
The tmTNF-α and IL-18 ROC curve for distinguishing between the CS and inflammatory response. The ROC curves of **a** tmTNF-α and **b** IL-18 during the differentiation between inflammatory response stage (*n* = 24) and CS stage (*n* = 24).

The pathological MOF process involves normal inflammatory responses and CS. In the MOF mouse model, the serum cytokine concentrations were high 4 h after LPS/D-gal injection, but the liver and kidney tissues were less damaged, which was consistent with the characteristics of a normal inflammatory response and was considered the inflammatory reaction stage. After 6 h, the serum cytokine concentrations were high, and the liver and kidney tissues were severely damaged, while the liver and kidney function were noticeably abnormal, which was consistent with the characteristics of CS, and was regarded as the CS stage. The ROC curve results showed that tmTNF-α differentiated between inflammatory responses and CS with an AUC value of 0.96 (95% CI, 0.92–1.00), a sensitivity of 89.29%, and a specificity of 89.29% (Fig. 4a), while the IL-18 AUC value was 0.63 (95% CI, 0.48–0.77) with 40.00% sensitivity and 85.19% specificity (Fig. 4b). The AUC value of the tmTNF-α significantly exceeded that of IL-18in distinguishing between inflammatory responses and CS. Compared with the inflammatory reaction stage, the serum cytokines in the CS stage were statistically significant, except for IL-18, while TNF-α, INF-γ, IL-4, and IL-6 displayed no statistical importance. It is suggested that tmTNF-α is superior to other serum cytokines in distinguishing between inflammatory responses and CS.

**Fig. 5:**
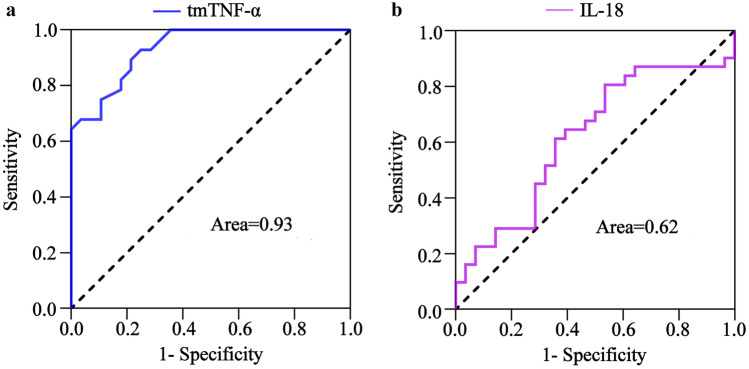
The tmTNF-α and IL-18 ROC curves for distinguishing between the live and dead mice. The ROC curve of **a** tmTNF-α and **b** IL-18 when differentiating between the dead (*n* = 28) and surviving mice (*n* = 28).

To further demonstrate the diagnostic value of tmTNF-α in CS, the AUC values of tmTNF-α and IL-18 were calculated to distinguish the live and dead mice. The results showed that tmTNF-α differentiated the live and dead mice with an AUC value of 0.93 (95% CI, 0.88–0.99), 90.14% sensitivity, and 80.00% specificity (Fig. 5a), while IL-18 exhibited an AUC value of 0.62 (95% CI, 0.47–0.76), a sensitivity of 80.65%, and a specificity of 46.43% **(**Fig. 5b). These results suggest that tmTNF-α displays potential diagnostic value for the disease severity caused by CS.

### Combining tmTNF-α and the Traditional Marker, CRP, can Improve Their Value for CS Diagnosis

**Fig. 6:**
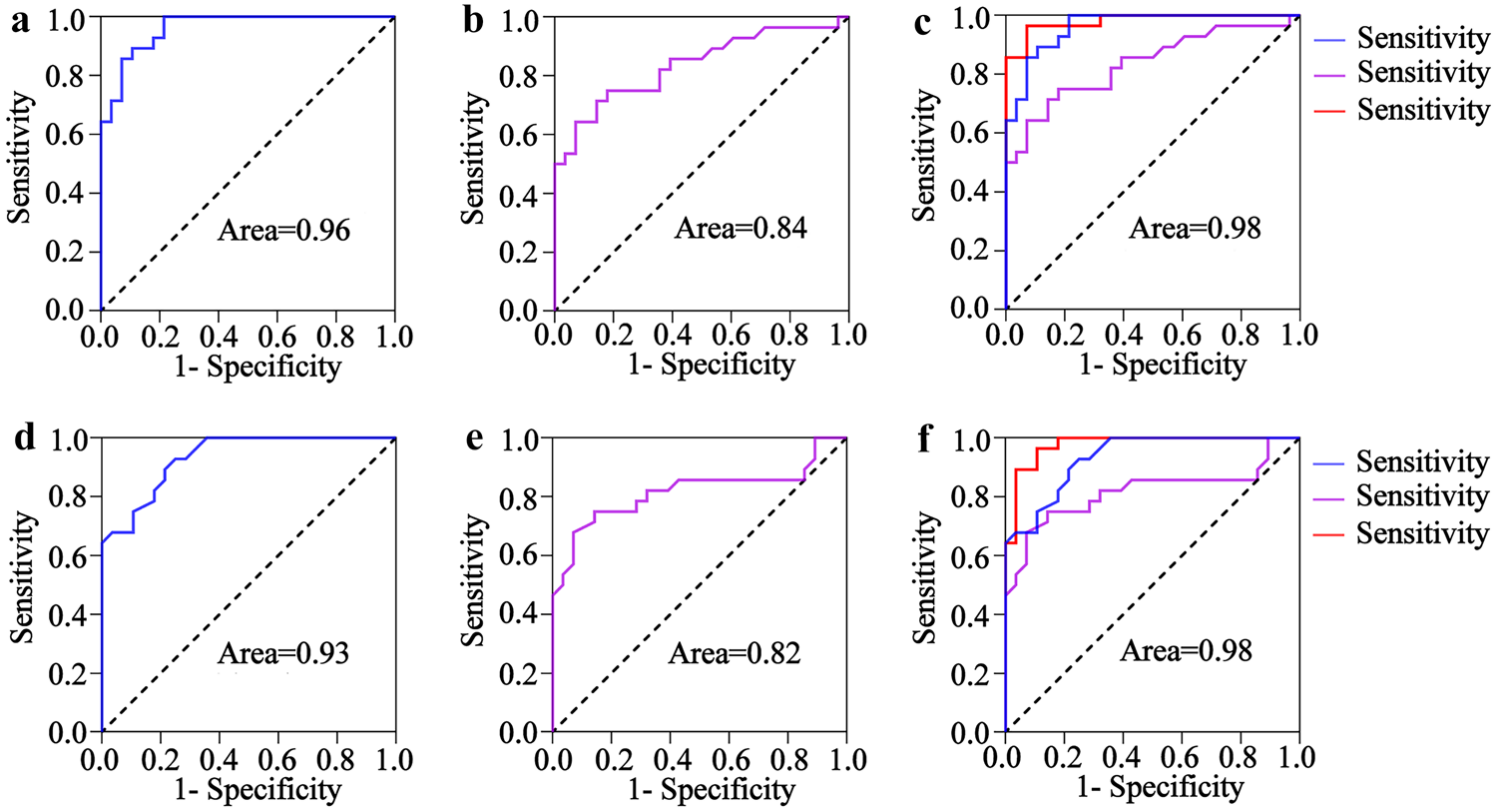
The ROC curve of the tmTNF-α and CRP combination for diagnosing CS. The ROC curves of the **a** tmTNF-α, **b** CRP, and **c** tmTNF-α and CRP combination for distinguishing between the inflammatory response and CS (*n* = 24). The ROC curves of the **d** tmTNF-α, **e** CRP, and **f** tmTNF-α and CRP combination for distinguishing between the dead and surviving mice (*n* = 28).

In clinical diagnosis, CRP is a standard indicator of the disease severity caused by CS. This study assessed the value of tmTNF-α combined with CRP for diagnosing CS. The results indicated that CRP differentiated between CS and inflammatory responses with an AUC value of 0.84 (95%CI, 0.73–0.94), 64.29% sensitivity, and 92.86% specificity (Fig. 6a), which was lower than tmTNF-α alone (Fig. 6b). The tmTNF-α and CRP combination substantially improved the diagnostic efficiency (AUC = 0.98, 95% CI, 0.95–1.00), while the sensitivity and specificity were 92.86% and 92.86%, respectively (Fig. 6c). Similarly, when comparing the live and dead mice, the CRP displayed an AUC of 0.82 (95% CI, 0.70–0.94), 67.86% sensitivity, and 92.86% specificity (Fig. 6d), which was lower than tmTNF-α alone (Fig. 6e). Furthermore, the diagnostic ability of the tmTNF-α and CRP combination was significantly improved, with an AUC value of 0.98 (95%CI, 0.94–1.00), 89.29% sensitivity, and 89.29% specificity (Fig. 6f).

## DISCUSSION

No single definition of CS is commonly accepted, while there is disagreement about how these disorders differ from an appropriate inflammatory response [3]. However, studies have shown that CS, in addition to high serum cytokine levels, is often accompanied by organ dysfunction and histopathological damage, which are closely related to the occurrence of acute liver failure, MOF, and sepsis [15–17]. In recent years, research has indicated that severe Ebola, avian influenza, SARS coronavirus, and novel coronavirus infections are also associated with the occurrence of CS [18, 19]. However, a reliable method for diagnosing CS is still lacking.

CS is closely associated with the exacerbation of acute liver failure [20, 21]. Liver injury induced by LPS/D-gal is a common liver failure model, presenting similar clinical symptoms to patients with liver failure. In addition to liver tissue damage and abnormal liver function, it is often accompanied by hepatic encephalopathy, lung injury, and renal injury [22, 23]. This study indicated that the blood TNF-α at 2 h after LPS/D-gal injection was significantly higher than the normal control but decreased to normal levels at 6 h. However, the tmTNF-α expression differed from the blood TNF-α, which was relatively low at 2 h and 4 h after injection but was substantially higher at 6 h, increasing with the deterioration of the damaged liver and kidney tissues. Common clinical markers for CS diagnosis include IL-6, IFN-γ, IL-4, and IL-18 [24–28]. Here, changes in the blood levels of these cytokines were different from those in tmTNF-α, which, despite their high levels, were not consistent with the severity of liver and kidney tissue injury. The expression levels of these cytokines did not reach a maximum in the most severe liver tissue damage cases. Except for IL-18, no significant differences were evident in the serum cytokines between 6 and 4 h after LPS/D-gal injection or between the live and dead mice. Significant differences were evident between the tmTNF-α expression levels at 6 h and 4 h after injection, as well as between the survival and death rates of the mice, suggesting that tmTNF-α displayed potential for diagnosing CS.

ROC curve analysis is a commonly used statistical method in clinical diagnostic tests [29, 30]. The diagnostic ability of test indexes can be directly judged according to the AUC value. To clarify the diagnostic ability of tmTNF-α, this study calculated the AUC value of tmTNF-α for CSwhileusingIL-18 as a control. The liver and renal tissue damage were mild 4 h after LPS/D-gal injection, with slightly abnormal liver and renal function, which was used as the normal inflammatory reaction stage. However, 6 h after this treatment, the liver and renal tissue damage were severe, while the functionality of these organs was highly abnormal, representing the CS stage. ROC curve analysis showed that the tmTNF-α AUC value was significantly higher than IL-18, suggesting that tmTNF-α could effectively distinguish between the inflammatory response and CS. Furthermore, this study evaluated the AUC value of tmTNF-α in distinguishing between the dead and surviving mice to assess its role in mitigating the disease severity caused by CS, indicating that the AUC value was significantly higher than IL-18. This suggests that the tmTNF-α expression level can effectively diagnose the disease severity resulting from CS.

Although CRP is a non-specific marker for diagnosing CS [31–33], its specificity and sensitivity levels do not meet clinical needs [34]. Combined with CRP, the tmTNF-α increased significantly compared to the value of CRP alone in differentiating inflammatory responses from CS, as well as in distinguishing between live and dead mice. The ROC curve analysis showed that CRP differentiated CS from inflammatory responses with an AUC value of 0.84 (95%CI, 0.73–0.94), while the tmTNF-α and CRP combination significantly increased the diagnostic value with an AUC value of 0.98 (95% CI, 0.95–1.00). Similarly, the AUC value of CRP was only 0.82 (95% CI, 0.70–0.94) in differentiating between the surviving and dead mice, while the combined CRP displayed a significantly higher AUC value of 0.98(95%CI, 0.94–1.00). These results imply that tmTNF-α can improve the diagnostic efficiency of CRP.

This study indicates that the tmTNF-α expression in the peripheral blood neutrophils can be used to diagnose CS and assess its severity. However, at present, it is only limited to animal experiments. It is unclear whether the expression of neutrophils in the peripheral blood of patients with MOF involving CS is increased and whether the expression level is related to disease severity. Moreover, whether the tmTNF-α expression in the peripheral blood neutrophils of patients with different degrees of CS display similar results requires further investigation.

## Supplementary Information

Below is the link to the electronic supplementary material.

Supplementary file1 (DOCX 4799 KB)
