# Supplementary material for: Transmembrane TNF-α as a Novel Biomarker for the Diagnosis of Cytokine Storms in a Mouse Model of Multiple Organ Failure
Source: Inflammation. 2022 Sep 14;46(1):359–69. doi: 10.1007/s10753-022-01738-6 (PMC9473472; doi:10.1007/s10753-022-01738-6)

**SUPPLEMENTARY MATERIAL**

**Supplementary Fig. 1** The dynamic liver histopathology and liver function changes in the MOF model mice. **(a)** The liver histopathology at different times after LPS/D-gal injection. The **(b)** serum ALT and **(c)** AST levels at different times after LPS/D-gal injection(n=7). The control group was compared with the treatment group at different times after LPS/D-gal injection.*indicates P<0.05,and ****indicates P<0.0001.

**
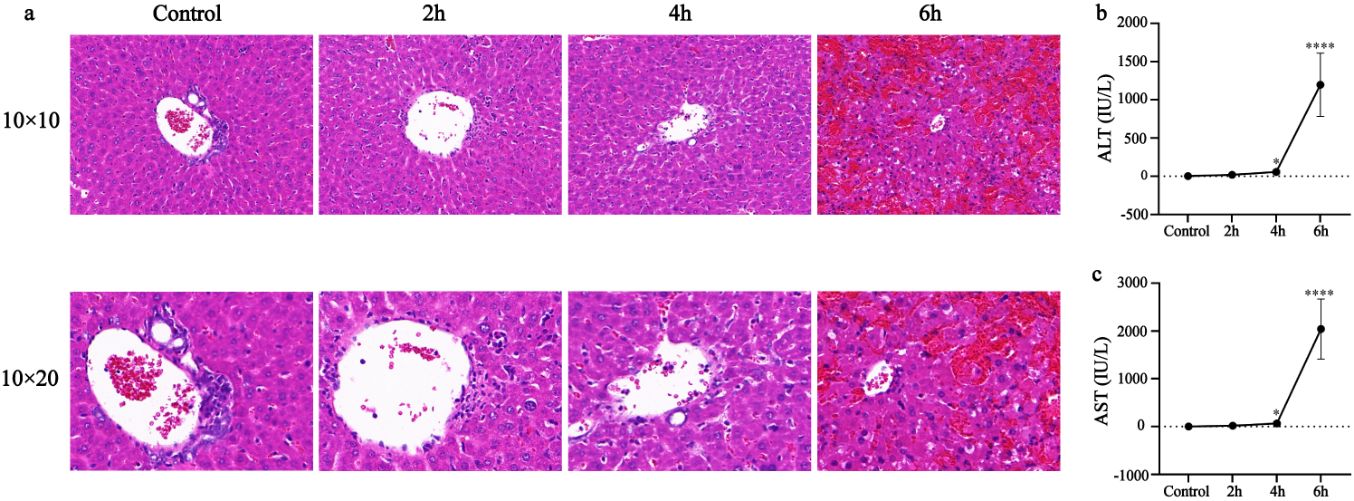
**

**Supplementary Fig. 2** The dynamicrenal histopathology and renal function changes in the MOF model mice. **(a)** The renal histopathology at different times after LPS/D-gal injection(n=7). The **(b)** serum Crand **(c)** CysC levels at different times after LPS/D-gal injection. The control group was compared with the treatment group at different times after LPS/D-gal injection.*indicates P<0.05, and ****indicates P<0.0001.


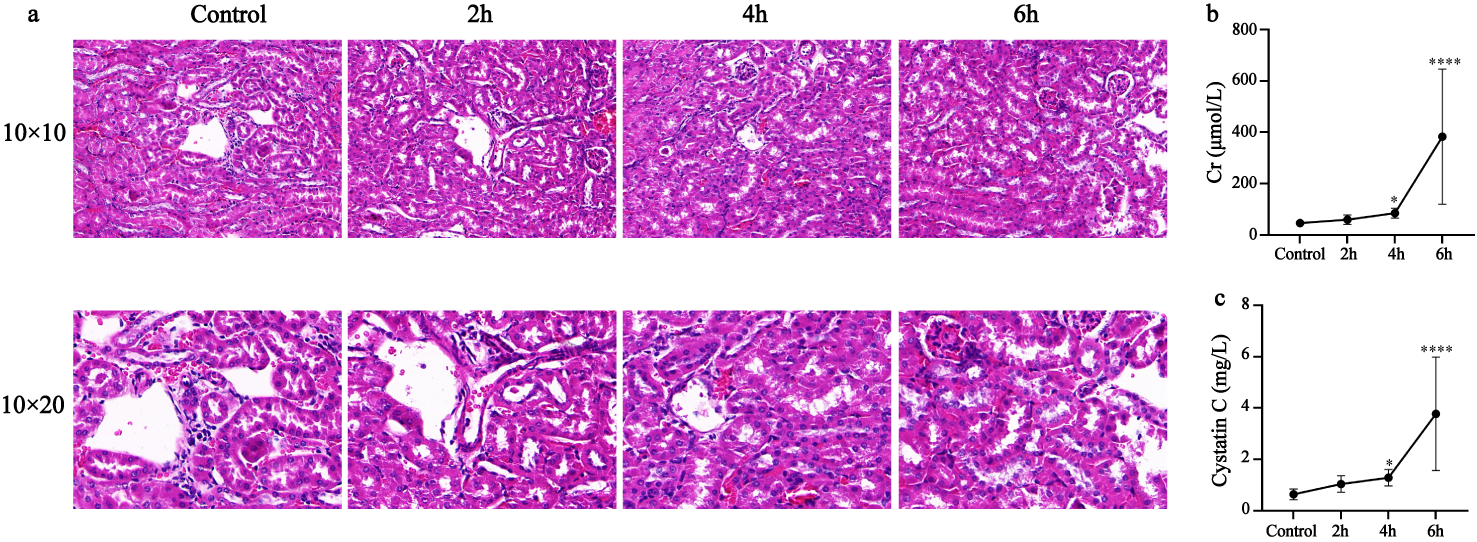

Supplement: Supplementary file 1 — Supplementary file1 (DOCX 4799 KB) [file 10753_2022_1738_MOESM1_ESM.docx]
